# The Latest Decalogue

**Published:** 1984-01

**Authors:** A. H. Clough


### Miscellanea

### THE LATEST DECALOGUE

The couplet printed in capitals is well known and frequently quoted, the remainder of the poem is less well known and readers may like to have the context from which it comes.

Thou shalt have one God only; who
Would be at the expense of two?

No graven images may be
Worshipped, except the currency.

Swear not at all, for, for thy curse
Thine enemy is none the worse.

At church on Sunday to attend
Will serve to keep the world thy friend.

Honour thy parents; that is, all
From whom advancement may befall.

THOU SHALT NOT KILL;
BUT NEED'ST NOT STRIVE
OFFICIOUSLY TO KEEP ALIVE.

Do not adultery commit;
Advancement rarely comes of it.

Thou shalt not steal; an empty feat,
When its so lucrative to cheat.

Bear not false witness; let the lie
Have time on its own wings to fly.

Thou shalt not covet; but tradition
Approves all forms of competition.

The sum of all is, thou shalt love
If anybody, God above.

At any rate shall never labour
More than thyself to love thy neighbour.

A. H. Clough (1819–61)

